# Does the principle of minimum work apply at the carotid bifurcation: a retrospective cohort study

**DOI:** 10.1186/1471-2342-11-17

**Published:** 2011-08-24

**Authors:** Richard J Beare, Gita Das, Mandy Ren, Winston Chong, Matthew D Sinnott, James E Hilton, Velandai Srikanth, Thanh G Phan

**Affiliations:** 1 Stroke and Aging Research Group, Department of Medicine, Monash University, Australia; 2 Murdoch Childrens Research Institute, Royal Melbourne Hospital, Melbourne, Australia; 3 Department of Radiology, Monash Medical Centre, Melbourne, Australia; 4 CSIRO Mathematics, Informatics and Statistics, Melbourne, Australia; 5 Menzies Research Institute, University of Tasmania, Hobart, Australia; 6 Stroke Unit, Monash Medical Centre, Melbourne, Australia

## Abstract

**Background:**

There is recent interest in the role of carotid bifurcation anatomy, geometry and hemodynamic factors in the pathogenesis of carotid artery atherosclerosis. Certain anatomical and geometric configurations at the carotid bifurcation have been linked to disturbed flow. It has been proposed that vascular dimensions are selected to minimize energy required to maintain blood flow, and that this occurs when an exponent of 3 relates the radii of parent and daughter arteries. We evaluate whether the dimensions of bifurcation of the extracranial carotid artery follow this principle of minimum work.

**Methods:**

This study involved subjects who had computed tomographic angiography (CTA) at our institution between 2006 and 2007. Radii of the common, internal and external carotid arteries were determined. The exponent was determined for individual bifurcations using numerical methods and for the sample using nonlinear regression.

**Results:**

Mean age for 45 participants was 56.9 ± 16.5 years with 26 males. Prevalence of vascular risk factors was: hypertension-48%, smoking-23%, diabetes-16.7%, hyperlipidemia-51%, ischemic heart disease-18.7%.

The value of the exponent ranged from 1.3 to 1.6, depending on estimation methodology.

**Conclusions:**

The principle of minimum work (defined by an exponent of 3) may not apply at the carotid bifurcation. Additional factors may play a role in the relationship between the radii of the parent and daughter vessels.

## Background

There has been recent interest in the role of carotid artery anatomy, geometry and hemodynamic factors in the pathogenesis of carotid artery atherosclerosis [1-6]. The anatomy and geometry at the carotid bifurcation within the same individuals [1,7] and between the sexes [3] vary greatly. The anatomy [7,8] and geometry [5] of the carotid bifurcation have large influence on vortex flow at the carotid sinus. These studies support observations that plaques form preferentially at such sites as carotid artery bifurcation (extracranial site) and the carotid artery siphon (intracranial site), rather than randomly in the carotid artery. This may provide an explanation for the asymmetrical nature of carotid artery stenosis [9] within the same individual despite exposure to the same factors [10]. In addition to this, there may be a role for interplay between anatomy, hemodynamic shear forces and traditional risk factors.

This work on carotid artery geometry has been inspired by the writings of Murray in 1926 [11] and Rossitti and Lofgren in 1993 [12,13]. A law on harmonisation of the vascular dimensions to minimize total energy cost by balancing energy lost to shear stress (frictional force) and the energy required to sustain the total blood volume (metabolic cost) throughout the vascular tree originated with the work of Murray in the 1920s and is known as the principle of minimum work [11] [see additional file 1]. The influence of this can be seen in the scaling law in biology describing how nutrients are transported through branching fractal network of vessels [14] and the scaling law of vascular trees such as the coronary tree [15]. In simple terms, this law predicts a cubic relationship (*n *= 3) between the radii of the parent and daughter vessels. Murray made several assumptions: 1- blood flow is non-pulsatile, has parabolic laminar flow profile and exerts constant shear stress on the arterial wall throughout the vascular tree, 2- the vessel is straight and rigid, and 3-the blood flow is described by Poiseuille's equation for flow in tubes [11]. The implication is that almost 94% of the frictional losses can be saved by doubling the radius of the tube while keeping length and other variables constant [16]. Rossitti and Lofgren examine the principle of minimum work in the intracranial internal carotid artery (ICA) to explore the importance of vascular dimensions on aneurysm formation. These investigators proposed that the vascular dimensions of the cerebral arteries follow the principle of minimum work [12] and that the bifurcations of the cerebral arteries appear to be optimized to avoid increased hemodynamic stresses [13]. These postulates have influenced recent works on hemodynamic factors in the development of carotid artery atherosclerotic disease [2] and intracranial aneurysms [17,18].

Recent studies provide evidence suggesting that the principle of minimum work applies at branching points several orders away from the aorta [13,17]. While the principle of minimum work may apply at the intracranial portion of the carotid artery, its applicability at the extracranial carotid artery bifurcation has not been tested [12]. In this study, we evaluate whether the bifurcation dimensions of the extracranial carotid artery follow this principle of minimum work. An understanding of this issue may shed light on the potential role of anatomy and geometrical factors in the development of carotid artery atherosclerosis.

## Methods

### Participants

This study involved patients who had CT angiography at our institution from 2006 to 2007 who had attended the Stroke and Vascular clinics. The Southern Health Ethics committee approved the study

### Imaging Protocol

Axial CT angiography scans were performed on a fourth generation CT Light speed scanner (General Electric Medical Systems, Milwaukee, WI) using a test bolus to determine the time of maximal contrast arrival in the artery. The field of view covered the aortic arch to the circle of Willis. The images have voxel resolution of 2 mm × 0.5 mm × 0.5 mm. The CT imaging uses a tube voltage of 120 kV, an effective current of 200 mA and 75 ml of intravenous contrast delivered through a power injector at 3 ml/sec.

### Segmentation

The method for segmentation of carotid arteries has been described previously [19]. In brief, a set of marker points was required to define the object of interest. Two marker points were used for each of the CCA, ICA and ECA and a sketch of the arterial tree was computed automatically using minimal cost paths. This sketch was used to initialize a watershed transform which produced a three dimensional mask of the arterial tree. In a validation study, the segmentation showed an intra-class correlation of 0.96 compared to manual segmentation by an expert neuroradiologist.

### Measurements of arterial radii and area

**Figure 1:**
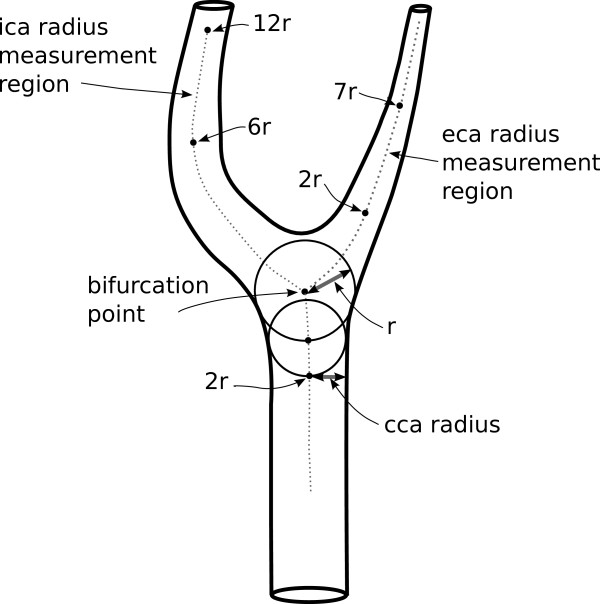
**Schematic diagram of a carotid artery bifurcation**. The parent artery (CCA) divides into two branches, ICA and ECA. Radius of CCA is measured at a location 2 sphere radiuses from the bifurcation point. Radii of the ICA and ECA are medians of radii measured at points between 6r - 12r and 2r-7r from the bifurcation point respectively. This approach avoided the ICA sinus and reduced influence of irregularities and artifacts due to ECA bifurcation.

Measurement of vessel radii and cross sectional area were derived from the centreline, or skeleton, of the segmented artery [20]. Two methods were used to estimate the radius. The first (method A) measured radius of the artery at every point on the centreline by computing the radius of the maximally inscribed sphere at each point [4]. In the second approach (method B), the centreline represented the flow axis of the artery and was used to compute the cross sectional area of the artery perpendicular to the direction of flow. Because some arteries do not have circular cross sections, we derived an equivalent radius from the cross sectional area. The two methods are equivalent for arteries with circular cross sections. Method A more closely approximates the approach used in clinical measurement of artery size, but will tend to underestimate cross sectional area when the shape is not circular. Method B was therefore included to ensure that underestimates of artery size due to non-circularity did not bias estimates of the exponent in the power law. The locations at which radii were measured were based on methods introduced by Thomas et al [4] that compute locations based on parameters of individual arteries. The CCA radius was measured at a distance of 2 radii from the bifurcation point (Figure 1)[4]. The ICA radius was estimated by taking the median of measures between 6 and 12 radii from the bifurcation point to avoid the carotid sinus. The ECA radius was estimated using the median of measures between 2 and 7 radii from the bifurcation point, avoiding artifacts due to ECA bifurcation.

### Determining the exponent of the power law (Analysis)

The principle of minimum work can be stated as

(1) $$r_{CCA}^{n}=r_{ICA}^{n}+r_{ECA}^{n}$$

where *r_CCA _*is the radius of the common carotid artery (CCA), *r_ICA _*is the radius of the internal carotid artery (ICA) and *r_ECA _*is the radius of the external carotid artery (ECA). The value for the exponent *n *derived by Murray using the energy minimization approach is 3. A brief derivation of this power law is provided in the additional file 1. To test the applicability of the energy minimization design principle to the carotid bifurcation, we solved Equation 1 for each bifurcation using numerical root finding methods. We also used nonlinear regression to estimate the optimum exponent for our study subjects. In addition, we evaluated the average change in ICA radius necessary to satisfy the power law. Nonlinear regression was performed using a Gauss-Newton algorithm with the *nls *function in the R statistics package [21]. Root estimation was performed using the *zeroin *Netlib algorithm [22] provided by the *uniroot *function in R.

Murray's model assumed straight arteries; hence we performed the analysis in a subset of patients with non-tortuous arteries, defined as tortuosity value less than 0.1. The tortuosity is defined as (L/D)-1, where L is the length along centreline and D is the Euclidean distance between two end points of the artery concerned [4].

## Results

A subset, 45/178, of the complete dataset with no carotid artery stenosis was used. The mean age of the subset was 56.9 ± 16.5 years and 26 were male. The prevalence of vascular risk factors in the subset was: hypertension 48%, smoking-23%, diabetes-16.7%, hyperlipidemia-51%, ischemic heart disease-18.7%. Fifteen of 45 subjects had a left ICA with tortuosity < 0.1 while 16 had a right ICA with tortuosity < 0.1. Radius ratios and bifurcation area ratios are summarised in Table 1.

**Table 1:** Artery radius ratios and standard deviations for arteries and bifurcation area ratio

Artery size measurement method	ECA/CCA	ICA/CCA	ECA/ICA	Bifurcation area ratio
A	0.61 (0.087)	0.67 (0.074)	0.92 (0.15)	1.28 (0.13)

B	0.65 (0.10)	0.68 (0.08)	0.96 (0.15)	1.33 (0.15)

**Figure 2:**
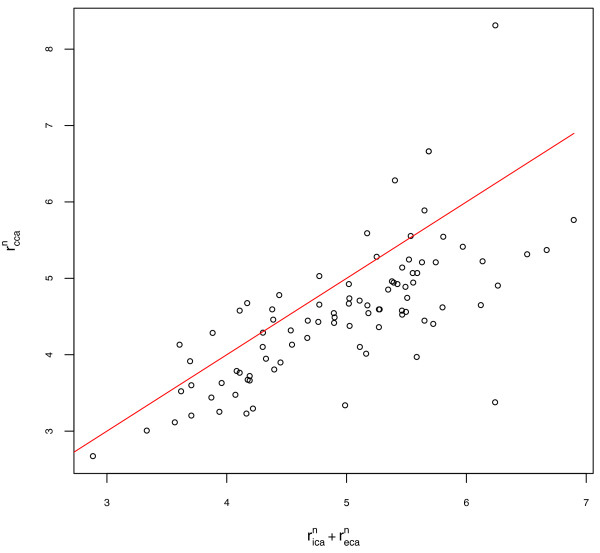
**Quality of fit for nonlinear regression**. r_ica_^n ^+ r_eca_^n ^vs r_cca_^n ^for all patients using artery size measurement method A and result of nonlinear regression (n = 1.32).

**Figure 3:**
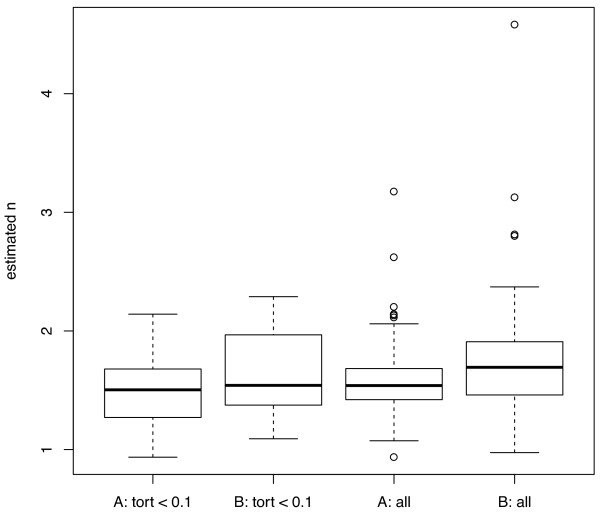
**Exponent values, n, for individual bifurcations for 45 patients**. Ranges of individual exponent values estimated for all patients (45) and patients with low tortuosity (31) using methods A and B for artery size measurement.

**Figure 4:**
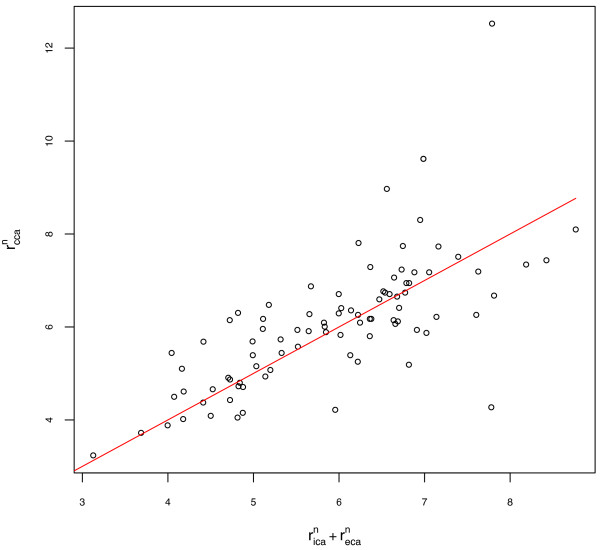
**Quality of fit for using mean of individual estimates**. r_ica_^n ^+ r_eca_^n ^vs r_cca_^n ^for all patients using artery size measurement method A and result of mean of individual estimates (n = 1.58).

No significant difference was observed between the left and right ICA radius measures or the left and right exponent values (paired t-tests, p = 0.5 and p = 0.4 respectively). Left and right arteries are grouped for all subsequent results. The exponent for the power law relating the radius of parent and daughter arteries, estimated for the cohort, ranged from 1.23 to 1.35, depending on the measurement method and the level of tortuosity. The results obtained using measurement methods A and B and for the two tortuosity categories are summarised in Table 2. The quality of fit for the nonlinear regression for measurement method A is illustrated in Figure 2. The spread of estimated values within the test population was large, and is illustrated using box and whisker plots of exponent values for individual bifurcations in Figure 3. The mean of the individual values of n estimated using root-finding methods was 1.58 for method A. The quality of fit assuming a value of n = 1.58 is illustrated in Figure 4.

**Table 2:** Estimated exponents and 95% confidence interval for all patients and arteries with low tortuosity using artery size measurements A and B

Artery size measurement method	Tortuosity < 0.1	All subjects
A	1.23 (1.09-1.34)	1.32 (1.24-1.40)

B	1.35 (1.20-1.46)	1.30 (1.13-1.42)

## Discussion

In this analysis of the extracranial carotid bifurcation, we have found that the value of the exponent, *n*, in the power law describing the sizes of the parent and daughter arteries is between 1.2 and 1.6, depending on estimation method, rather than the value of 3 predicted using Murray's energy minimization argument. The estimates obtained for individual bifurcations, using numerical root finding methods, and for the subject populations, using nonlinear regression, were consistent, with very few subjects having exponents close to 3. The differences in estimate between nonlinear regression and numerical root-finding approaches remained despite removal of possible outliers and tests with alternative solvers and are most likely attributable to the weighting used during nonlinear fitting. Figures 2 and 4 suggest that the estimate based on mean of individual estimates is more appropriate in this dataset, with the line of best fit appearing better centred on the data. Only small differences in estimated exponent values were observed when different methods for estimating artery radius were used, or when the analysis was restricted to a subset of the study population with less tortuous arteries. The different methods for estimating artery diameter are likely to have a bigger impact in diseased arteries where artery cross-sections are less circular. The values obtained for *n *for individual arteries varied considerably and were not centred on either 2 (corresponding to preservation of cross sectional area) or 3. Changes in ICA radius necessary to satisfy the power law while holding CCA and ECA radius constant were of the order of 20% for *n *= 2 and 40% for *n *= 3. These finding suggest that additional factors such as variation in flow velocity during cardiac cycle, shape of the carotid sinus and vessel elasticity contribute to the relative sizes of arteries at the carotid bifurcation.

The population had artery radius ratios and bifurcation area ratios between those previously reported in [23] and [24].

### Methodological limitations

In this study, the measurements of arterial radii were taken from the arterial lumen due to the use of CT angiography images. The radiological contrast agent in CT angiography depicts the lumen rather than the exterior of the arterial wall. However, this use of luminal radii is similar to the measurement of radii in Rossitti and Lofgren's studies and is unlikely to have affected the analysis [12,13]. Our use of three-dimensional angiography data made it possible to measure the cross sectional area of arteries perpendicular to the central axis. This measurement is likely to be more difficult to perform consistently using conventional two-dimensional angiography data, potentially leading to errors in arterial radii measures. Such errors may explain dramatically different exponents for the power law obtained for the intracranial artery - 2.9 (two-dimensional angiogram data) [12] and 1.7 (three-dimensional angiogram data) [17].

Our finding that the exponent of the power law is much less than 2 occurs despite efforts to perform the analysis in relatively straight arteries that are appropriate for Murray's model and using radius derived from cross sectional area. Despite removal of subjects with carotid artery stenosis, it is possible that the use of CT angiography images from patients attending vascular and stroke clinics rather than from the community may have affected our results. Previous findings of exponents less than 2 in studies using three-dimensional techniques [17] provide some re-assurance that this choice of subject did not greatly bias our results.

### Implications for Murray's Law

The exponent of the power law at the carotid bifurcation in this study is different from intracranial component [12,17]. This finding supports the suggestion that the exponent of the power law is not constant throughout the vascular tree [25]. In line with this possibility, investigators suggested that the exponent of the power law is 2 in the major branches close to the aortic arch, 2.5-3 in coronary, 2.9 in MCA bifurcation, to 3 in arterioles [17,25-27]. In-vivo measurements of wall sheer stress (WSS) [25] show that the assumption of constant WSS throughout the body made by Murray is unrealistic and hence the variation in power law exponent is not surprising. An exponent of 2 corresponds to preservation of area and therefore constant flow velocity into and out of the bifurcation. Exponents greater than 2 imply a decrease in flow velocity leaving the bifurcation while exponents less than 2 imply an increasing flow velocity leaving the bifurcation.

Murray's law does appear to be useful in describing size of small vessels (canine arteries with radius of 0.159 cm and capillaries, radius 3.5 × 10^-4 ^cm) as illustrated in the original calculations. The law also successfully predicts the sizing of human coronary arteries (mean radius 1.44 mm) [27] and the MCA (mean radius 1.2 mm) [17]. However the law does not apply in larger arteries.

These variations suggest that any energy minimization argument used to explain vascular size throughout the body and across species should include more terms and be more complicated than the shear stress and volume argument proposed by Murray. Possibilities include energy terms relating to energy loss due to reflections at bifurcations that may become more significant in areas where the blood flow is more pulsatile or where the flexibility of vessel walls interacts significantly with the nature of flow.

## Conclusions

This study examined the relationship between radiuses of arteries at the extracranial carotid bifurcation using CTA imaging in 45 subjects. The power law of Murray [11] did not apply to this data. More complex terms are therefore needed if minimization of energy arguments can be used to describe carotid bifurcation geometry.

## Competing interests

The authors declare that they have no competing interests.

## Authors' contributions

RB developed segmentation and measurement methods, performed analysis and helped draft manuscript. GD developed measurement methods, performed analysis and helped draft manuscript. MR and WC carried out artery segmentation and validation of segmentation methods. MS and JH provided fluid dynamics expertise and helped draft manuscript. VS and TP conceived the study, participated in its design and coordination, participated in analysis and helped draft the manuscript. All authors read and approved the final manuscript.

## Pre-publication history

The pre-publication history for this paper can be accessed here:

http://www.biomedcentral.com/1471-2342/11/17/prepub

## Supplementary Material

Additional file 1 **brief derivation of Murray's law**. Click here for file
