# Supplementary material for: Does the principle of minimum work apply at the carotid bifurcation: a retrospective cohort study
Source: BMC Med Imaging. 2011 Aug 24;11:17. doi: 10.1186/1471-2342-11-17 (PMC3178471; doi:10.1186/1471-2342-11-17)
Supplement: Additional file 1 — brief derivation of Murray's law. [file 1471-2342-11-17-S1.DOCX]

# Murray’s Law – a brief derivation

A brief description based on the original derivation by Murray is given here. The total power required to sustain a regulated flow of blood through the vessel can be written as:


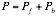
 (1)

where P_f_ is the amount of power required to drive the blood flow and P_b_ is the metabolic power needed to maintain the flow. From Poiseuille’s equation, the first term can be written as:


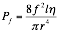
 (2)

where r is the radius and *l* is the length of artery, η is the viscosity of blood, f is the flow. The total power P, involved in operating a section of artery is given by:

$P=\frac{8f^{2}l\eta}{\pi r^{4}}+bl\pi r^{2}$ (3)

where b is the metabolic coefficient. Now, the maximum economy of work can be obtained by equating the first derivative of P with respect to r as equal to zero. This leads us to the following equation:


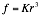
 (4)

where K is a constant.

Now, from the principle of continuity of flow, the flow in mother artery should equate the sum of flows in the daughter arteries. Thus,

$$f_{0}=f_{1}+f_{2}$$

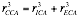
(5)
